# Innovations in ion-selective optodes: a comprehensive exploration of modern designs and nanomaterial integration

**DOI:** 10.3389/fbioe.2024.1397587

**Published:** 2024-08-19

**Authors:** Ahmed Barhoum, Yaser Alhashemi, Yomna M. Ahmed, Mahmoud S. Rizk, Mikhael Bechelany, Fatehy M. Abdel-Haleem

**Affiliations:** ^1^ NanoStruc Research Group, Chemistry Department, Faculty of Science, Helwan University, Cairo, Egypt; ^2^ Chemistry Department, Faculty of Science, Cairo University, Giza, Egypt; ^3^ Ministry of Interior, Farwaniya, Kuwait; ^4^ Institut Européen des Membranes (IEM), UMR 5635, University Montpellier, ENSCM, CNRS, Place Eugène Bataillon, Montpellier, France; ^5^ Gulf University for Science and Technology, GUST, Hawally, Kuwait; ^6^ Department of Chemistry, College of Science, Imam Mohammad Ibn Saud Islamic University (IMSIU), Riyadh, Saudi Arabia

**Keywords:** optodes, ionophores, indicator dyes, polymer membranes, nanomaterials, wearable optodes, smartphone-based optodes, disposable paper-based optodes

## Abstract

In recent years, ion-selective optodes (ISOs) have remarkably progressed, driven by innovative modern designs and nanomaterial integration. This review explored the development of modern ISO by describing state-of-the-art strategies to improve their sensitivity, selectivity, and real-time monitoring capacity. The review reported the traditional membrane based-optodes, and investigated the latest research, current design principles, and the use of essential components, such as ionophores, indicator dyes, polymer membranes, and nanomaterials, in ISO fabrication. Special attention was given to nanomaterials (e.g., quantum dots, polymer dots, nanospheres, nanorods and nanocapsules) and particularly on how rare earth elements can further enhance their potential. It also described innovative ISO designs, including wearable optodes, smartphone-based optodes, and disposable paper-based optodes. As the pursuit of highly sensitive, selective, and adaptable ion sensing devices continues, this summary of the current knowledge sets the stage for upcoming innovations and applications in different domains (pharmaceutical formulations, medical diagnosis, environmental monitoring, and industrial applications).

## Graphical Abstract

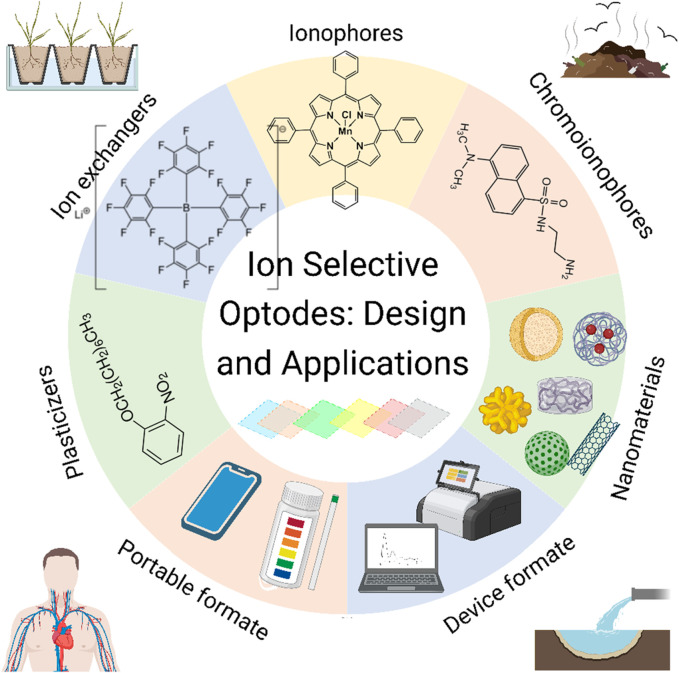

## 1 Introduction

Ion-selective optodes (ISOs), established as optical chemical sensors since the 1970s, have undergone significant modernization driven by contemporary design principles (Maduraiveeran et al., 2018). ISOs are used to identify and quantify specific ions in various matrices, such as pharmaceutical formulations, biofluids, and environmental samples. ISOs rely on a selectively crafted sensing platform where the choice of materials, including ionophores, polymers, indicator dyes, nanomaterials and conductive materials, is crucial for sensitivity, selectivity and overall performance (Du and Xie, 2021). These materials, carefully selected and combined, enhance ISO potential for many different applications. ISOs can be categorized on the basis of their ion specificity and sensing mechanisms into different classes, such as pH optodes, metal-complexing optodes, solvatochromic optodes, and quantum dot (QD)-based optodes. ISOs take advantage of different processes, such as ion-exchange, metal-ligand interactions and fluorescence modulation, for precise ion detection (Hu et al., 2018; Merl et al., 2022). ISO specificity through molecular recognition can be obtained by integrating ionophores, which are molecules that bind to specific ions, and chromoionophores, which undergo reversible color changes upon binding to ions (Merl et al., 2022; Nussbaum et al., 2022). Signal transduction mechanisms, based on different methods (e.g., fluorescence quenching, absorbance modifications and luminescence changes), play a crucial role in translating ion-binding events into detectable signals, thus providing distinct signal outputs linked to the ion concentration (Merl et al., 2022).

Nanomaterial integration into ISOs has introduced unique properties, enhanced sensitivity and enabled real-time monitoring. It also reflects recent trends in sensor technology. The use of nanomaterials, including nanoparticles and nanowires, alongside polymers and paper substrates, has expanded ISO versatility (Saranchina et al., 2021). Advanced techniques, such as microfluidics, nanolithography, and layer-by-layer assembly, have recently emerged for providing precise control of the optode design and for further improving their sensitivity and selectivity (Wu and Qin, 2014; Saranchina et al., 2021). ISOs offer multiple advantages, such as rapid response times, real-time measurements, portability, and the potential for miniaturization (Cui et al., 2022). However, recent challenges involve addressing issues related to their long-term stability, reproducibility, and the impact of environmental conditions on the sensor performance. Additionally, there is a growing emphasis on enhancing ISO biocompatibility for biomedical applications. Despite the progress, standardized protocols and validation processes are still needed to ensure ISO reliability in different applications and settings. Moreover, ISO compatibility with portable devices, such as smartphones, has significantly expanded their utility for on-site and point-of-care (POC) measurements (Yadav et al., 2022). Progress in microfabrication techniques is driving ISO miniaturization and integration into smart platforms, including wearable skin sensors, showcasing the dynamic evolution of this field (Yadav et al., 2022).

This review shed light on the traditional membrane-based optodes, and comprehensively explored ISOs, guided by the overarching themes of innovative modern designs and integration of nanomaterials. It mentioned first the PVC-membrane optodes, its components, history, some reviews for this types, and mechanisms that obeyed by this type. This review, also, discussed the latest research and the incorporation of the basic components (ionophores, indicator dyes, polymer membranes) in ISO production. Notably, it focused on nanomaterials (QDs, polymer dots, nanospheres, nanorods and nanocapsules), with an emphasis on how rare earth elements can increase their performance. It then described the basic principles underlying microsphere- and nanosphere-based ISOs. This review also discussed groundbreaking ISO designs, including wearable optodes, smartphone-based optodes and disposable paper-based optodes. This review is a testimony of the researchers’ creativity and collaborative efforts that drive progress in this field. Their research is reshaping ISO landscape and opening the doors to a future filled with innovative possibilities. The ultimate objective of this review was to provide insights into ISO fabrication processes, analytical performances and potential applications for selecting the most appropriate and cost-effective architecture for the desired application.

## 2 Traditional membrane optodes and its theoretical bases

Polymer-based membranes play a crucial role in the functionality and performance of ISOs. Polyvinyl chloride (PVC) is the most preferred choice due to its chemical stability, flexibility, and compatibility with various plasticizers and ionophores, making it suitable for detecting ions like potassium, sodium, calcium, and chloride in clinical, environmental, and industrial applications (Bakker et al., 1997; Yaacob et al., 2024; Xie et al., 2012). Polyurethane (PU) membranes and hydrogels are valued for their high mechanical strength, biocompatibility, and good adhesion, making them ideal for biomedical applications and robust environmental sensors (Pedersen et al., 2015). Cellulose acetate-based membranes, known for its biodegradability and good film-forming properties, is used in disposable optodes for detecting ions in biological fluids and environmental samples (Strömberg et al., 2009). Each polymer offers unique advantages, enabling the development of customized ISOs for a wide range of applications.

Polymer membranes can be deposited on various supports such as glass, paper, and cellulose-based materials, each offering unique advantages. Glass provides a stable, inert, and non-porous surface, ensuring high reproducibility and durability with excellent optical clarity, ideal for precise and reliable measurements like detecting potassium and sodium in clinical samples (Heng et al., 2003). However, its rigidity and fragility limit its use in portable applications. Paper is inexpensive, flexible, and easy to handle, making it perfect for disposable sensors and field applications, with its porous nature enhancing analyte interaction and detection sensitivity, as seen in detecting heavy metals like lead and cadmium in water samples (Kassal et al., 2019a). Nevertheless, paper’s durability can be compromised by humidity and mechanical stress. Cellulose and its derivatives, like cellulose acetate, combine flexibility and biodegradability with reasonable durability, making them suitable for integration into microfluidic devices for portable, on-site analysis, such as pH sensing in biological fluids (Salmani et al., 2018; Morf et al., 1989). However, they are generally less stable than glass, potentially affecting long-term performance. Each substrate offers unique benefits tailored to specific applications, whether for laboratory precision, field testing, or portable device integration.

**FIGURE 1:**
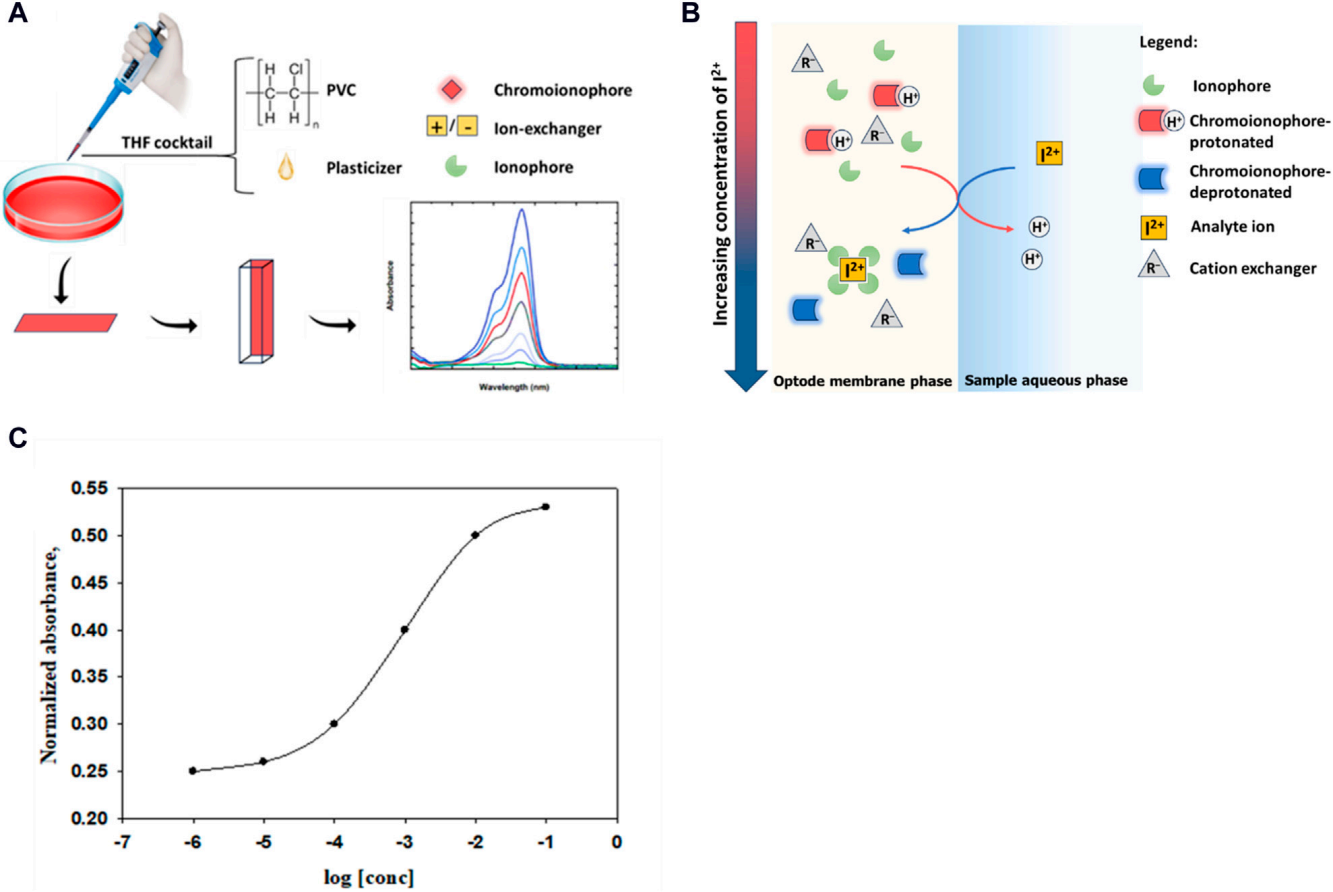
Different designs of ISOs: **(A)** Preparation of the membrane optode. **(B)** Response mechanism in chromoionophore-based membrane optodes to an analyte cation. **(C)** The sigmoidal shape of the calibration curve of the membrane selective optode.

PVC membrane optodes, or bulk optodes, are very often made of hydrophilic materials and including lipophilic indicator; it incorporates the ionophore, the chromoionophore and the ion-exchanger (Bühlmann et al., 1998); drop-casting for the cocktail containing the membrane components on glass and quartz substrates, as in Figure 1A, was applied and reported by several reviews for many years until the modification of ISOs by the following different modifiers (Bakker et al., 1997; Bühlmann et al., 1998; Hisamoto et al., 1997). The mechanism depends on the protonated and deprotonated forms of the indicator, that differ in their optical properties, thus making the change in protonation degree in the sensor recordable. The fundamental principle underpinning H^+^-chromoionophore-based optodes lies in the intricate engineering of the chromoionophore molecule itself. This careful design results in a molecule that exhibits reversible shifts in color in response to variations in proton concentration, which is related to analyte ion concentration (Xie and Bakker, 2022). Upon interaction with H^+^ ions, a reversible protonation process induces modifications in the chromoionophore’s electronic configuration, leading to observable changes in absorbance or emission properties, Equation 1.

$$I_{aq}^{+}+{\text{nL}}_{m}+R_{m}^{-}+{zCH}_{m}^{+}↔\;{zH}_{aq}^{+}+{IL}_{n,m}^{z+}+Z\;C_{m}+R_{m}^{-}$$ (1)

Where ${CH}_{m}^{+}$ and C_m_ represent the protonated and deprotonated forms of the chromoionophore in the membrane optode phase, L_m_ and ${IL}_{\;m}^{z+}$ represent the ionophore in the free and complexed states in the membrane, $R_{m}^{-}$ is the cation exchanger additive in the optode membrane phase, and $H_{a}^{+}and\;I_{a}^{+}$ represent the proton and the analyte cation in the aqueous solution; the exchange constant for the reaction of Equation 1, as reported by Bakker et al. (1997), can be expressed as in Equation 2:

$$K_{exch}^{IL_{n}}={\left(\frac{a_{H}\left[C\right]}{\left[{CH}^{+}\right]}\right)}^{z}\left(\frac{\left[{IL}_{\;n}^{z+}\right]}{a_{I}L^{n}}\right)$$ (2)

The response depends on the ratio between the activities of the analyte cation and the proton, Eq. 1. By the same method, an equation can be derived for the anion analyte, where the optode response depends on the product of the activities of the analyte anion and the proton, Equations 3, 4.

$${{C\;}_{m}+L\;}_{m}+H_{a}^{+}+I_{a}^{-}+↔{LI}_{m}^{-}+{CH}_{m}^{+}$$ (3)

The co-extraction constant which corresponds to this equilibrium is

$$K_{exch}^{IL_{n}}=\frac{{\left[CH\right.}_{m}^{+}][{LI}_{m}^{-}]\;}{\left[L_{m}\right]\;\left[C_{m}\right]}\;.\;\frac{1}{{a_{H^{+}}\;.a}_{I^{-}}}$$ (4)

With the assumption that the electroneutrality condition, the mass balance of ionophore and chromoionophore is verified, the proportion of the unprotonated form of the chromoionophore (α) can be expressed in Equation 5

$$\alpha =\frac{A_{p}-A}{A_{p}-A_{D}}$$ (5)

where $A_{p},A_{D,}$ represent the absorbance of the fully protonated and deprotonated chromionophores, and A is the absorbance at any equilibrium situation. The ideal sigmoidal calibration curve can be obtained from the relation between α and the activity logarithm of the analyte, Figure 1C. These chromatic variations are quantifiable through optical measurements, providing a direct and real-time representation of the analyte concentration level present in the analyzed sample, Figure 1B. Notably, the versatility of H^+^-chromoionophore-based optodes is exemplified by their seamless integration into various platforms, including polymeric, microfluidic devices, and wearable sensors (Brady et al., 2021). This adaptability enhances their potential for real-time, on-site monitoring, thus making them applicable across an array of scientific and industrial contexts.

It's important to acknowledge that while the inherent sigmoidal nature shapes the calibration curve, Figure 1C; deviations from this ideal pattern may be observed. Such deviations are particularly pronounced in cases where membrane optodes are manually prepared (Abdel-Haleem et al., 2022). Numerous instances of optodes have been documented, catering to various ion types, including monoions, polyions, and neutral species (Abdel-Haleem, 2016; Taha et al., 2022).

## 3 Novel materials for ion-selective optodes

Modern ISOs have significantly evolved in design and materials, capitalizing on the progress in materials science, nanotechnology, and fabrication techniques to enhance their sensitivity, selectivity, and overall performance. Notably, nanomaterials (nanoparticles, nanotubes, nanocomposites) are now integrated into ISOs to bring unique properties, such as higher surface area, tunable surface chemistry and enhanced mass transport, that improve the sensor performance (Barhoum et al., 2023a). For example, metal nanoparticles (gold or silver), functionalized with specific ionophores, are used to fabricate highly selective and sensitive sensors. QDs (i.e., semiconductor nanocrystals with size-dependent optical properties) are incorporated for multi-analyte sensing and improved signal transduction (Dubach et al., 2007a; Rahimi et al., 2019). The integration of smart polymers and responsive materials allows producing dynamic ISOs that undergo structural changes upon ion binding, providing real-time and reversible sensing assays. Stimulus-responsive polymers, such as hydrogels and molecularly imprinted polymers, enhance the ionophore selectivity and stability (Barhoum et al., 2023b). Microfabrication techniques allow fabricating miniaturized and multiplexed ISOs with microfluidic channels, lab-on-a-chip devices, and array formats for the simultaneous measurement of multiple analytes (i.e., real-time monitoring). These designs, which are particularly useful for clinical diagnosis, environmental monitoring and high-throughput screening, include ISOs tailored for detecting heavy metal ions, wearable sensors for on-body monitoring of physiological ions, and optodes integrated into lab-on-a-chip platforms for POC measurements. Membrane based-optode technique was the one of the best choices during the previous modifications using PVC, Nafion^®^, triacetyl cellulose or methacrylic-acrylic copolymers (Bühlmann et al., 1998; Absalan et al., 2010; Nurlely et al., 2022; Worlinsky et al., 2014); metals and carbonaceous nanomaterials, molecularly-imprinted polymers and other modifiers were incorporated into the PVC membrane (Abdel-Haleem et al., 2016; Worlinsky et al., 2014; Wiorek et al., 2022; Yaacob et al., 2024; Kłucińska et al., 2016).

### 3.1 Micro/nanosphere-based ion-selective optodes

Micro/nanosphere-based optodes, a forefront innovation in optical sensing, offer versatile and sensitive platforms for ion detection (Du et al., 2022). These optodes incorporate ion-selective components, such as ionophores and chromoionophores, on or within microspheres (i.e., small spherical particles from nanometers to micrometers in size). This enables their specific interactions with the target ions, leading to measurable optical changes. The higher surface area-to-volume ratio of micro/nanospheres enhances the optode binding capacity and sensitivity, facilitating precise detection even in complex sample matrices. The fabrication of microspheres relies mainly on emulsion polymerization, precipitation polymerization, or sol-gel methods and the use of polymers (e.g., polystyrene, poly(methyl methacrylate), Pluronic^®^ F-127 copolymer) or silica, due to their ease of synthesis, biocompatibility, and tunable properties. Once synthesized, microspheres must be functionalized to incorporate ionophores or chromoionophores using surface modification techniques, such as covalent attachment or physical adsorption. Carefully selected ionophores exhibit high selectivity for the target ions, enabling specific binding. The core of nanosphere or microsphere were made from different types of polymers such as poly (methyl-methacrylate), pluronic copolymer, polystyrene or others (Abdel-Haleem and Zahran, 2019; Zhai et al., 2015; Xie and Bakker, 2022).

The advantages of micro/nanosphere-based optodes, including ease of handling, high sensitivity, and potential for miniaturization, are major assets for many sensing applications. Lead-selective microspheres, embedded with ionophores, enable the quantification of lead in water samples. For instance, functionalization of microspheres (∼13 µm) with the ionophore ETH 5493 and the chromoionophore ETH 5418 gave high lead ion selectivity with a limit of detection (LOD) of 3 × 10^−9^ M at pH 5.7, suitable for rapid and sensitive detection of trace levels for environmental and biological applications (Telting-Diaz and Bakker, 2002). Polystyrene microspheres (0.8–2.4 µM in diameter) functionalized with the K^+^ ion ionophore valinomycin, as a proof of concept, were recently tested by Xie et al. for measuring K^+^ ions, with a linear response range from 10^–6^ to 10^–1^ M in pH 5.6 (Xie et al., 2014a). Guinovart et al. reported sulphate determination using polystyrene microspheres (0.8 µm size) that incorporated bisthiourea as ionophore and fluorescein octadecyl ether as chromoionophore (Guinovart et al., 2015). These functionalized microspheres exhibited high sensitivity (<60 nM) and LOD of 0.06 µM based on mass-extraction equilibrium. Abdel-Haleem and Zahran prepared polystyrene microspheres (0.2–0.4 µm in size) for salicylate determination at concentrations between 3 and 70 µM. They reported a LOD of 2.1 µM with high selectivity and reproducibility and also fast response time (seconds) (Abdel-Haleem and Zahran, 2019).

**FIGURE 2:**
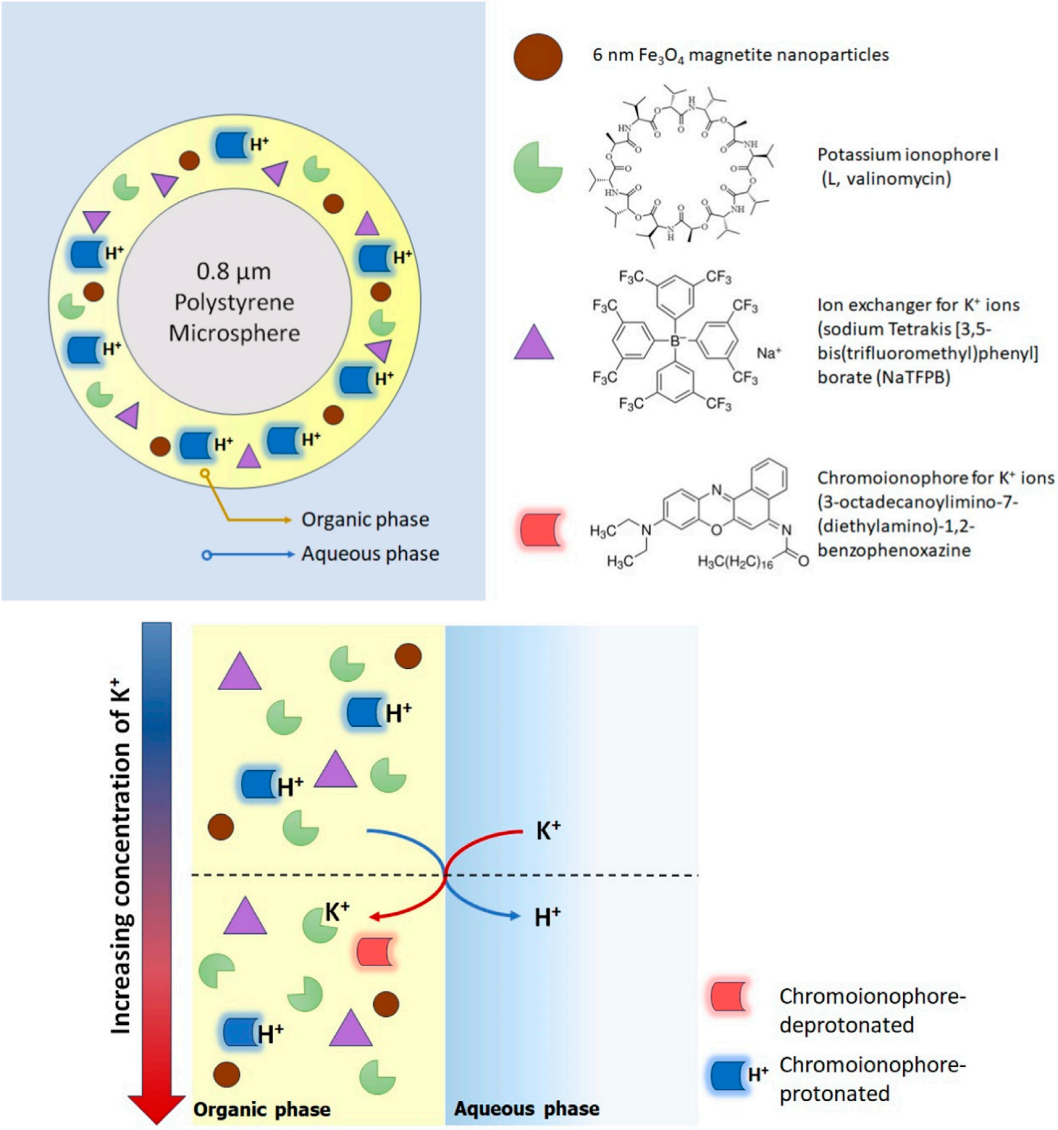
Illustration of reversible magnetic ion-selective colorimetric microsensors using surface-modified polystyrene beads (Apichai et al., 2020).

For the first time, surface-modified polystyrene beads (0.8 μm in diameter) have been used to create magnetic ion-selective colorimetric microspheres (Apichai et al., 2020). This involves combining essential ISO components (chromoionophore, ion-exchanger, and ionophore) with magnetic nanoparticles. These components are skillfully adsorbed onto the polystyrene particle surface through a straightforward mixed solvent approach. Using fluid circulation and magnetic attraction, these reversible micro-sensors can be concentrated at a specific point where they can be visualized with a digital camera. Hue signals extracted from the captured images facilitate the quantification of the chromoionophore protonated and deprotonated forms, forming the core of the optode response. These magnetic micro-sensors exhibited exceptional selectivity for K^+^ ions at concentrations between 10^−6^ M and 10^–2^ M, with a swift response time (t_99_ < 2.6 ± 0.5 min above 10^–5^ M) (Figure 2). It should be noted that in these microsensors, solvatochromic dyes (used as pH-independent transducers) did not give good results (Apichai et al., 2020). Although polystyrene microspheres offer high stability and ease of preparation, their limited sensitivity affects the detection limit of the optical sensor. Moreover, the performance of the optode depends on the size of the formed microsphere.

**FIGURE 3:**
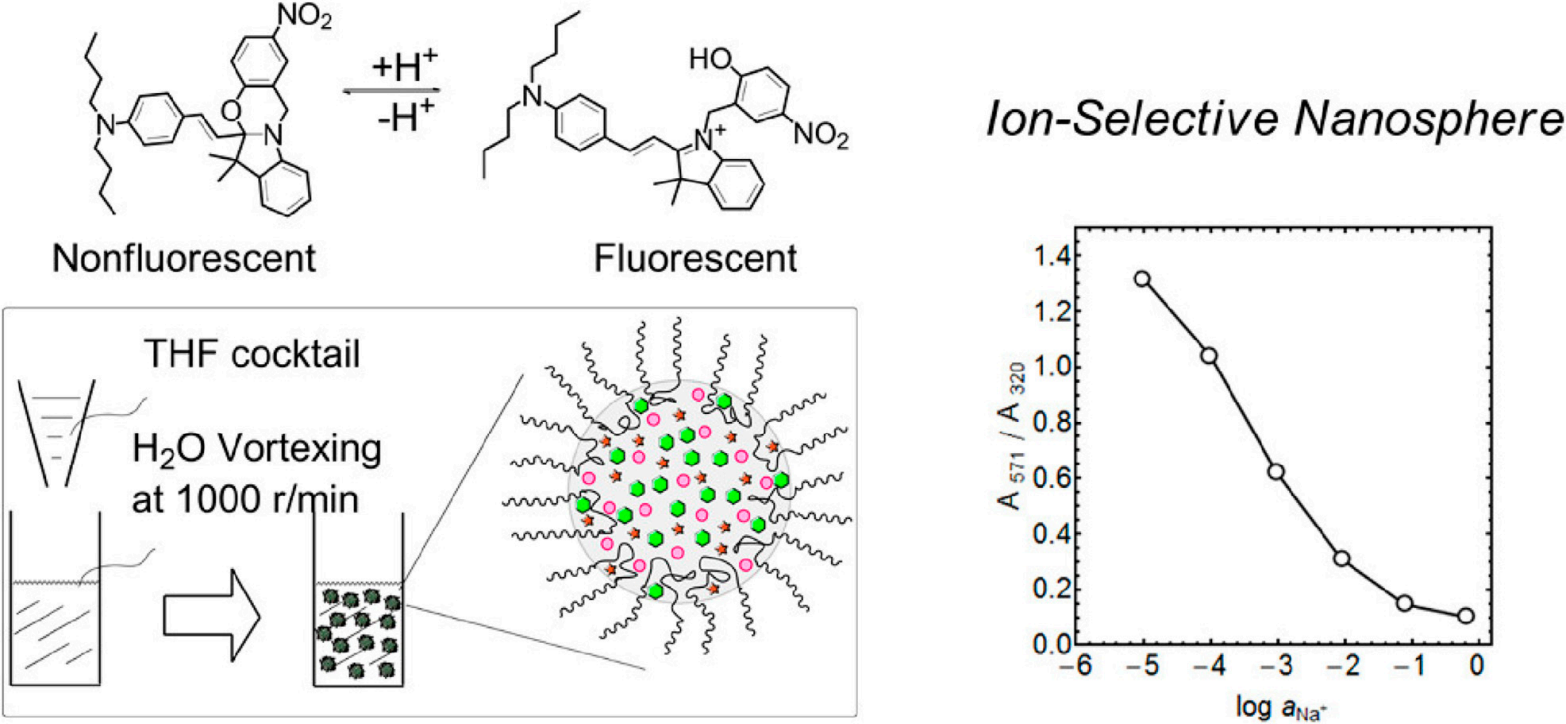
Illustration of the process to fabricate ultrasmall fluorescent ion-exchanging nanospheres that incorporate the oxazinoindoline chromoionophore and sodium-selective ionophores. The fabrication involves integrating a non-ionic and biocompatible surfactant, Pluronic^®^ F-127 (F127), into the solution. F127 has a central hydrophobic poly(propylene oxide) chain flanked by two hydrophilic poly(ethylene glycol) (PEG) chains (Xie et al., 2013). THF, tetrahydrofuran. Copyright © Open Access (ACS, 2013).

To overcome the limitations of polystyrene, other materials are also used in micro/nanosphere fabrication such as surfactant polymers. Xie et al. (2013) described a straightforward method for producing monodisperse ultrasmall nanospheres (40 nm in diameter) tailored for ion-selective applications. This involves injecting a tetrahydrofuran solution that contains the sensing components into deionized water and incorporating the biocompatible surfactant Pluronic^®^ F-127 (F127) into the solution. F127 hydrophobic poly(propylene oxide) chains interact with other hydrophobic sensing components (a sodium ionophore and oxazinoindoline or a Nile blue derivative dye as chromoionophore) to form the core structure, while F127 hydrophilic poly(ethylene glycol) (PEG) chains act as a surfactant, preventing nanosphere merging (Figure 3). These ultrasmall nanospheres, stabilized by F127, exhibit stability, and versatility, are easy to fabricate and are robust tools for detecting inorganic ions at very small concentrations. The authors showed that by incorporating a neutral sodium ion (Na^+^)-selective ionophore into the nanospheres, the sensitivity and selectivity for Na^+^ were improved. Indeed, they could determine Na^+^ concentration in commercial mineral water samples. These nanospheres are candidates for wide applications in chemical biology and environmental science.

Nanosphere-based ISOs have been extensively investigated to determine their mechanisms (Ferris et al., 2018) and applications in solvatochromic dyes (Zhai et al., 2015), complexometric titration (Zhai et al., 2015), QDs (Du et al., 2020), and other areas (Aldea et al., 2020). Zhai et al. (2015) used 210-nm nanospheres containing a solvatochromic dye as an indicator and F-127 as a substrate for complexometric titration. This method offers a sharp endpoint, increased versatility, and eliminates the need for pH control. These nanospheres were tested to determine calcium (Ca^2+^) ion concentration in mineral water and showed precision and high accuracy. In another study, nanospheres (20–50 nm in size) with F-127 were used to determine the acid dissociation constant (pKa) of chromoionophores (Xie X. et al., 2016). Additionally, F-127-based nanospheres were tested in innovative applications for determining poly-ions, such as heparin and protamine, showing high sensitivity and selectivity compared with traditional optode designs (Soda et al., 2021). The authors employed dinonylnaphthalenesulfonate as hyperpolarizing lipophilic phase to polarize the X^3+^ solvatochromic dye. This allowed the reliable quantification of these analytes in human plasma at low heparin concentrations (0–0.8 U/mL). Overall, the use of F127 in nanosphere optical sensors offers advantages such as stabilization of nanoparticles, efficient encapsulation of hydrophobic molecules, high solubility in water, low toxicity and biocompatibility.

However, despite their numerous advantages, micro/nanosphere-based ion-selective optodes also have certain limitations compared to other detection methods (Xie and Bakker, 2022). One significant limitation is the complexity and cost associated with their fabrication process, which often involves specialized equipment and techniques such as emulsion polymerization or sol-gel methods. This can make their production more time-consuming and resource-intensive compared to simpler sensor designs. Additionally, the need for surface functionalization to incorporate ionophores or chromoionophores adds another layer of complexity to the fabrication process. Furthermore, while micro/nanosphere-based optodes offer enhanced sensitivity, they may still be susceptible to interference from complex sample matrices, limiting their applicability in the real-world. While micro/nanosphere-based ion-selective optodes offer significant advantages in terms of sensitivity and versatility, their fabrication complexity and susceptibility to interference may pose challenges in practical applications.

### 3.2 Quantum dot-based ion-selective optodes

QD-based optodes are a cutting-edge development in ion-selective sensing and provide a highly sensitive platform for detecting various ions and molecules (Xie R. et al., 2016). QDs are nanoscale semiconductor particles with unique optical and electronic properties due to quantum confinement effects and are ideal for designing optodes with enhanced sensitivity and selectivity. In the traditional optode design, quantum dots were encased within the PVC-polymer matrix; the mechanism depended on the pH-chromoionophore and an inner-filter effect (Dubach et al., 2007b); upon analyte cation extraction from the aqueous phase to the optode phase, a proton from the chromoionophore was dissolved in the aqueous phase, causing color change of the polymer film. As the absorbance of the colored film increases, the attenuation of the fluorescence of the quantum dots increases, which can be recorded and related to analyte concentration (Dubach et al., 2007b). In other optodes, ion-specific recognition elements are immobilized on the QD surface, allowing selective interactions with the target ions. Upon binding, the QD optical properties, such as fluorescence emission and absorbance, undergo distinct changes that can be measured to determine the target ion concentration (Zhu et al., 2015). For instance, boron- and nitrogen-co-doped graphene-QDs in a QD-based optode were used for sensitive mercury (Hg) detection in real water samples (LOD of 6.4 nM Hg^2+^) (Liu et al., 2020) for environmental pollution monitoring. Similarly, QD-based optodes have been developed for detecting Ca, which is crucial in many biological processes, by using carboxyl-coated CdSe/ZnS QDs (20 nm diameter) functionalized with a Ca-detecting aptamer and gold nanoparticles (LOD = 3.77 p.m. Ca^2+^) (Ghosh et al., 2020). The selective binding of Ca ions induced changes in the fluorescence signal, enabling the real-time monitoring of Ca dynamics in biological systems. Although this optical sensor enabled picomolar Ca ion detection, the overall sensor preparation is considered expensive and time-consuming.

**FIGURE 4:**
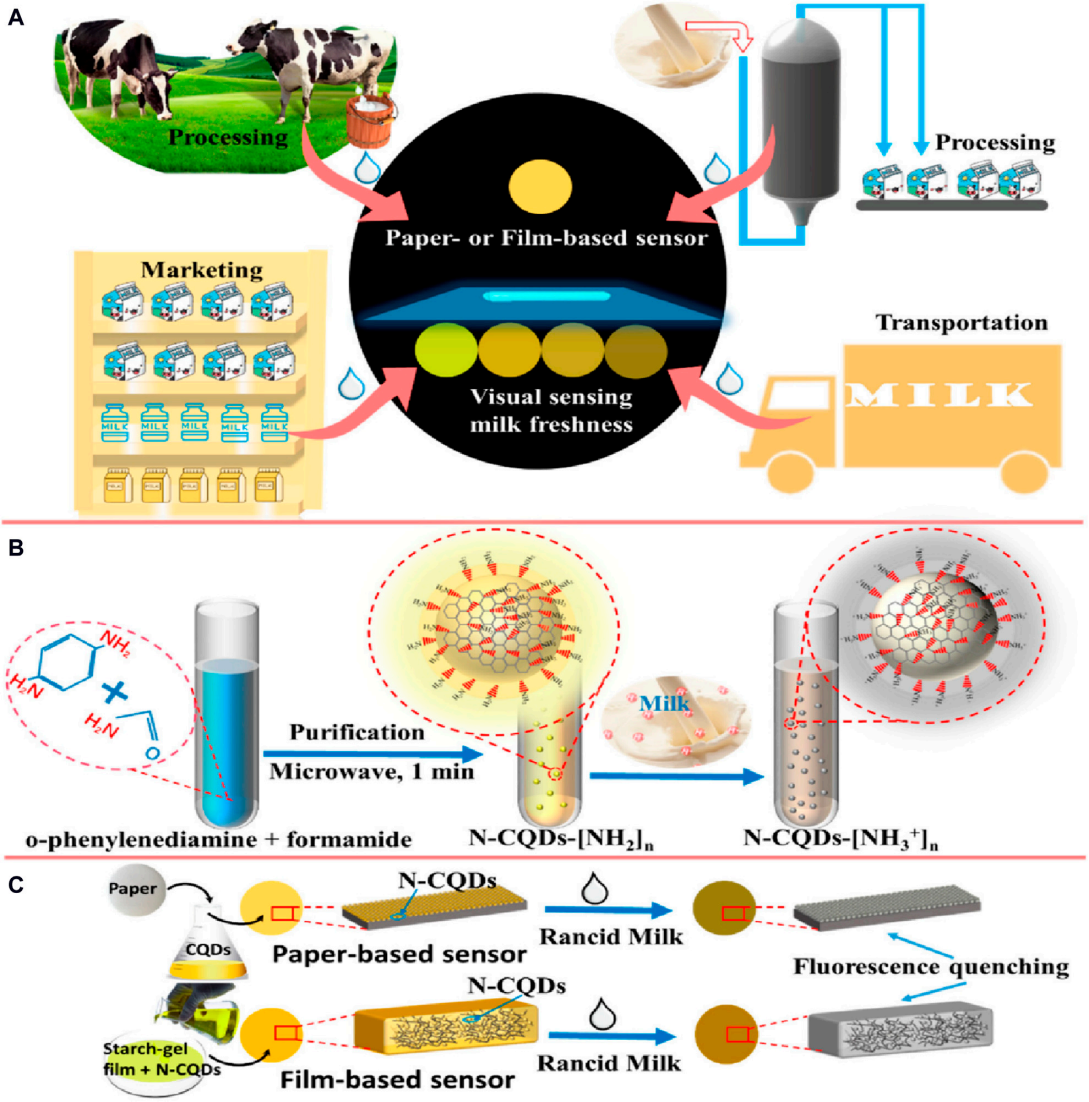
Visual sensing system for milk freshness monitoring using fluorescence sensors based on nitrogen-doped carbon QDs (N-CQDs): **(A)** Easy-to-use visual sensing device of milk freshness; **(B)** N-CQD fabrication; and **(C)** Fluorescence quenching of sensors by rancid milk (Hu et al., 2022). Copyright MDPI (2022).

QD-based ISOs have been applied in biosensing for medical diagnosis to detect specific biomolecules, such as glucose and proteins (Yan et al., 2017; Hu et al., 2018). For instance, a CdTe QD-glucose oxidase-based aerogel could detect glucose (linear concentration range of 1–12 mM) with a LOD of 0.135 nM. Another fluorescent sensor, nitrogen-doped graphene QDs/SiO_2_/mitoxantrone (78 nm diameter), was used for cytochrome C determination with a LOD of 0.11 µM. These performances have implications for POC diagnosis and disease monitoring. Nitrogen-doped carbon QDs (N-CQDs), a novel fluorescent material, can be used for food assessment, biological analysis, and environmental monitoring due to their exceptional optical qualities and biocompatibility. A recent study (Hu et al., 2022) described a user-friendly sensing system to assess milk freshness visually based on N-CQDs sensitive to acidity. The shift in fluorescence brightness, detectable by the naked eye, correlates with decreasing milk freshness (and thus increasing milk acidity), facilitating the development of a colorimetric card for rapid milk freshness assessment in 2 min and without pretreatment. This approach holds promise for sensitive, quick, and user-friendly monitoring devices of food quality and safety (Figure 4).

CdTe/CdS QDs, known for their high fluorescence quantum yield, are commonly employed as probes for sensing a single target. In a novel approach, Wei et al. (2020) introduced an optical sensing method based on CdTe/CdS QDs to simultaneously sense Fe^3+^, Cr_2_O_7_^2−^, and IO_3_^−^ by exploiting the different response mechanisms of Fe^3+^ and IO_3_^−^: photo-induced electron transfer and inner filter effect, respectively. These distinct mechanisms generated different responses that allowed differentiating between analytes, even at varying concentrations. The linear detection ranges were 5.0–100.0 μM for Fe^3+^, 20.0–140.0 μM for Cr_2_O_7_^2−^, and 1.0–80.0 μM for IO_3_^−^, with remarkably low LOD (4.1 μM, 9.7 μM, and 0.9 μM, respectively). The method was successfully tested with real samples, demonstrating its effectiveness for iodate and total iodine detection in table salt samples. This innovative multi-target detection strategy, based on single fluorescence from individual QDs, holds promise for enhancing sensing efficiency and opens avenues for diverse applications. However, QDs containing heavy metals have limited applications *in vivo* due to their toxicity. To overcome this problem, it is better to use polymer dots due to their biocompatibility.

Overall, QD-based ion-selective optodes offer unique optical and electronic properties, derived from quantum confinement effects, enhancing sensitivity and selectivity compared to traditional sensors (Naresh and Lee, 2021). QDs allow multiplexed detection of multiple ions simultaneously, crucial in complex sample matrices. Their high quantum yield and brightness enable detection in low concentrations, suitable for high-sensitivity applications. Additionally, QD-based optodes remain stable against photobleaching, ensuring reliability in continuous monitoring (Shamirian et al., 2015). Their small size and compatibility with surface modification techniques facilitate the integration of ion-specific recognition elements, promoting selective binding interactions and accurate detection. However, limitations exist. Certain QDs, especially those with heavy metals like cadmium or lead, pose potential toxicity risks, limiting their use in certain biological or environmental sensing contexts (Wu and Qin, 2014). Moreover, the synthesis and purification of QDs can be costly, hindering widespread adoption, particularly in resource-limited settings. Additionally, optimizing surface functionalization and stabilization for specific ion recognition may pose challenges, requiring specialized expertise. Despite these limitations, QD-based ion-selective optodes offer promising avenues for sensitive and selective ion detection in various applications.

### 3.3 Polymer dot-based ion-selective optodes

In polymer dot-based optodes, polymer dots (Pdots) are fused with ISOs to introduce luminescent nanoparticles composed of conjugated polymers (Ma et al., 2019; Du et al., 2020). Pdots have exceptional optical properties, including bright and tunable fluorescence, high photostability, and minimal photo-blinking. This combination has attracted considerable attention for its potential to revolutionize ion sensing (Sun et al., 2018; Ma et al., 2019). By integrating ionophores and chromoionophores onto the Pdot surface, the optode ion selectivity and responsiveness are improved. The interaction between ionophore and target ion induces changes in Pdot fluorescence, facilitating real-time and quantitative ion detection. Pdot unique optical features, such as narrow emission bands and resistance to quenching, enhance sensitivity and accuracy. Pdot-based optodes have been developed for Ca, K, Na, pH, and protein determination (Sun et al., 2018; Ma et al., 2019; Du et al., 2020). Their advantages include reduced interference from sample matrices, improved signal-to-noise ratio, and compatibility with complex biological systems. Importantly Pdots can be integrated into various sensing formats, including microfluidic devices, wearable sensors, and imaging probes.

**FIGURE 5:**
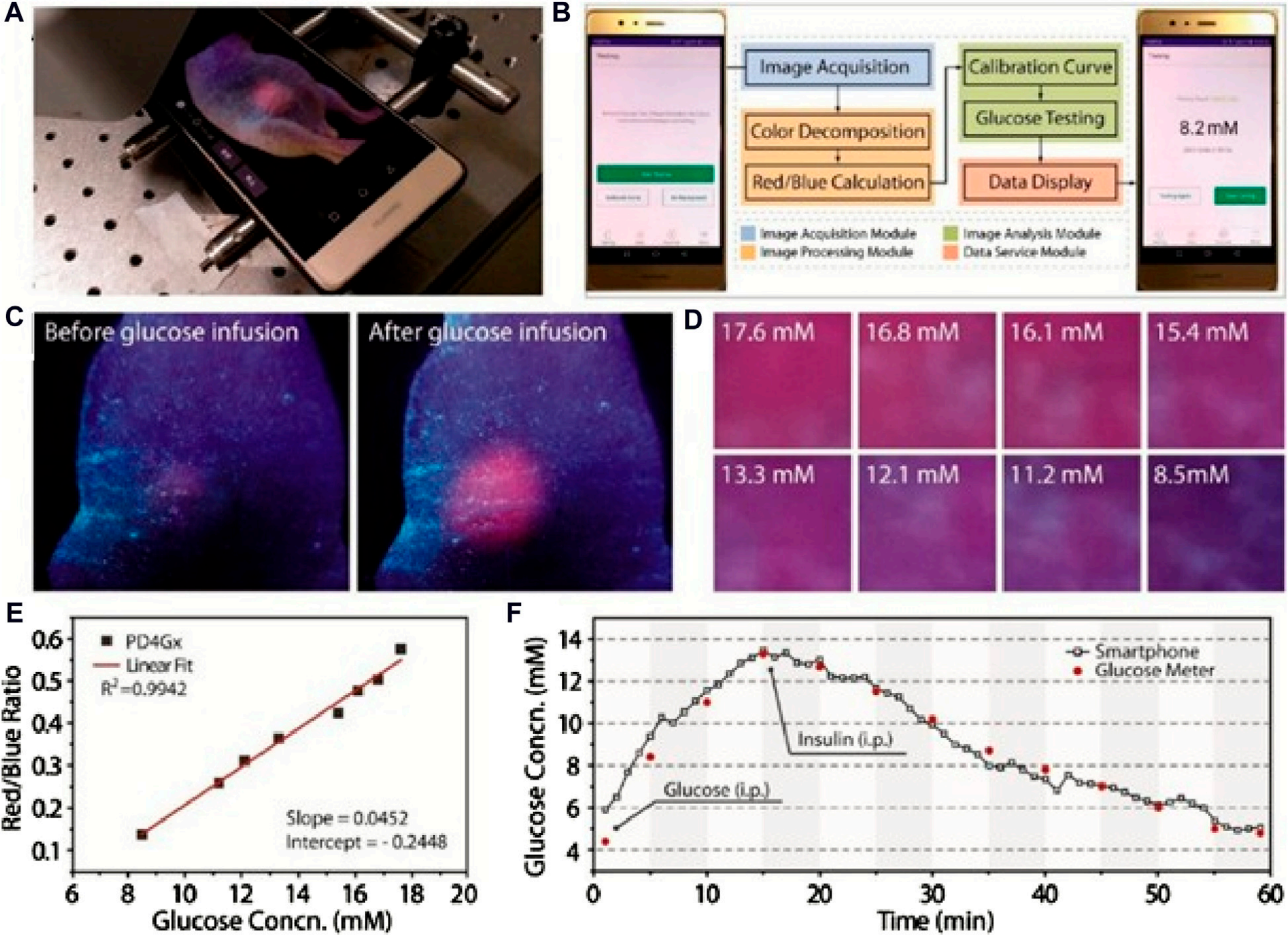
A polymer-dot transducer (PD4Gx) for wireless glucose monitoring with a smartphone. *In vivo* glucose monitoring in mice with the PD4Gx transducer and a smartphone: **(A)** Live glucose measurement. **(B)** Image decomposition for blood glucose monitoring. **(C)** True-color images before and after glucose administration. **(D)** Magnified images highlighting glucose concentration changes over time after glucose infusion. **(E)** Calibration curve showing the correlation between red/blue intensity ratio and glucose concentration (*R* ^2^ > 0.99), and **(F)** Real-time dynamic glucose monitoring with the implanted PD4Gx and a smartphone, including the fluctuations after glucose and insulin administration; red scattered points indicate glucose measurements in tail blood using a commercial glucose meter (Sun et al., 2018). Copyright © ACS (2018).

Polymer nanodot-based optodes, a subset of nanosphere-based optodes, use a poly(styrene)-graft-poly(ethylene oxide) copolymer as substrate (Yavitt et al., 2019). This copolymer rapidly self-assembles and is an effective template for various nanomaterials. Du et al. (2020) tested a nanodot-based optode for Na and K determination in blood samples. The nanodots had a size <25 nm (by cryogenic electron microscopy) and increased selectivity, by two orders of magnitude, compared with nanosphere-based sensors. The smaller size also increased the complexation constant between target ions and ionophores on the nanodot surface. Sun et al. (2018) developed a highly sensitive optical transducer for wireless glucose monitoring using smartphones. This setup combines oxygen-sensitive polymer dots with glucose oxidase, achieving precise glucose detection. Skillful Pdot design resulted in a one-order-of-magnitude sensitivity enhancement. Smartphone-captured images were used to discriminate between euglycemia and hyperglycemia. Moreover, real-time monitoring was demonstrated in mice in which a Pdot transducer had been implanted. The outcomes showed a linear response (*R* ^2^ > 0.99) for glucose concentrations in blood ranging from 6 to 18 mM, with a sensitivity of ∼60% per mM. Although smartphone-derived sensitivity was slightly lower compared with the spectroscopic results (152% per mM), these findings highlight the viability of this Pdot transducer combined with a smartphone for glucose monitoring (Figure 5).

Overall, Pdots optodes stand out as a promising method for ion detection, particularly due to their exceptional optical properties and improved ion selectivity. Compared to traditional ion-selective electrodes, Pdots offer significant advantages (Dubach et al., 2007a). These include bright and tunable fluorescence, high photostability, and minimal photo-blinking, providing superior optical characteristics crucial for accurate sensing. Additionally, by integrating ionophores and chromoionophores onto the polymer dot surface, these Pdots exhibit enhanced ion selectivity and responsiveness, allowing for real-time and quantitative detection. The interaction between the ionophore and target ion induces changes in polymer dot fluorescence, facilitating precise ion detection. Furthermore, the unique optical features of Pdots, such as narrow emission bands and resistance to quenching, enhance sensitivity and accuracy in ion detection applications (Dubach et al., 2007a). Despite these advantages, challenges such as complex synthesis and functionalization processes, limited ionophore compatibility, potential cost implications, and susceptibility to environmental interference may impact their widespread adoption.

### 3.4 Nanocapsule-based ion-selective optodes

Nanocapsule-based ISOs represent a breakthrough in ion sensing by combining nanocapsule encapsulation efficiency and ionophore selectivity (Kim et al., 2012; Kisiel et al., 2018). These nanometer-sized hollow structures, often made of polymers or lipids, protect the encapsulated ionophores, thus ensuring their stability and shielding them from interference. The interaction between ionophores and target ions induces detectable changes in the nanocapsule properties, providing the basis for optical signal detection and facilitating the precise quantification of the ion concentration. Nanocapsule-based ISOs can be used in many different applications, from metal ion detection (e.g., Ca and magnesium), to pH monitoring and analyte detection in complex samples (Maclin et al., 2015). Notably, Nile blue A-loaded porous nanocapsules exhibited sensitivity to pH changes with ±0.03 pH unit resolution. Encapsulation allows fine-tuning the optode selectivity, response time, and sensitivity. Copper ion-selective nanocapsules, with a linear response to a concentration range from 0 to 400 nM and LOD of 2.6 nM, are a good example of their potential (Vellaichamy and Periakaruppan, 2017).

**FIGURE 6:**
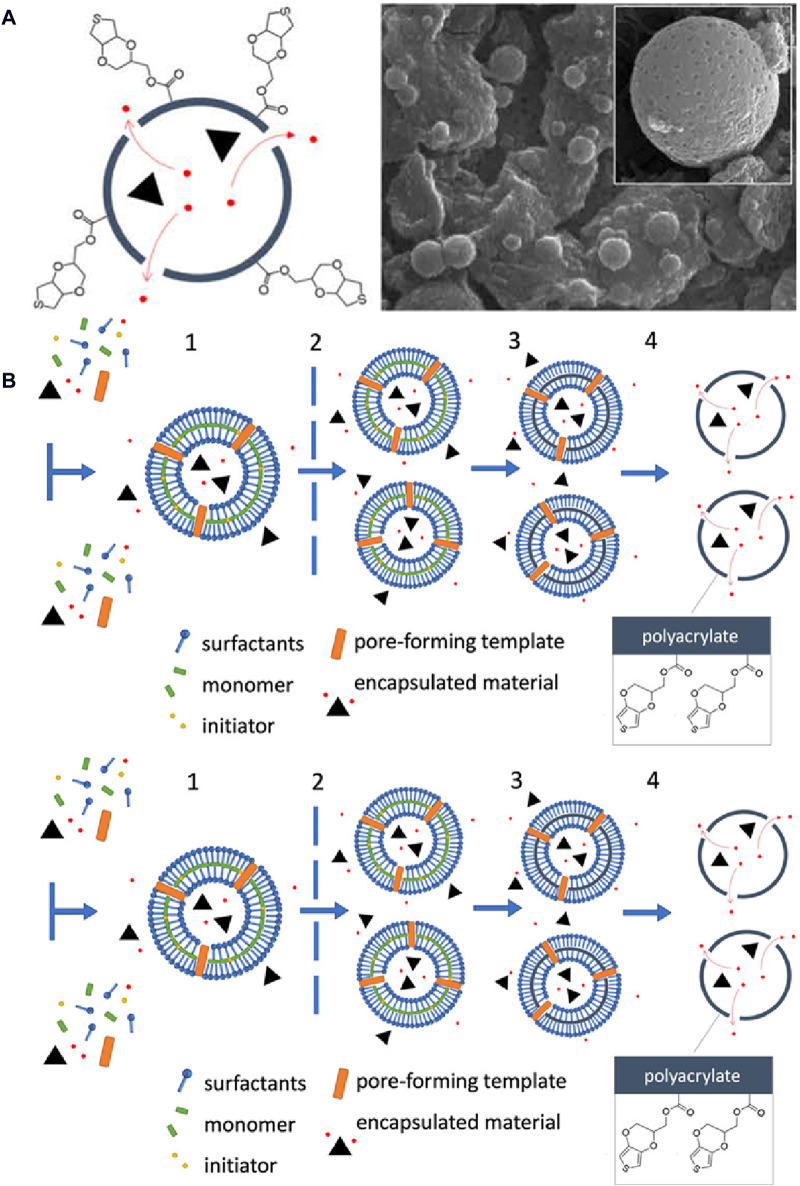
Deposition of 3,4-ethylenedioxythiophene (EDOT)-decorated hollow nanocapsules onto PEDOT films for optical and electrochemical sensing: **(A)** Schematic representation of a nanocapsule and a scanning electron microscopy image. **(B)** Overview of the synthesis steps for creating EDOT-decorated nanocapsules. (1) Anionic and cationic surfactants self-assemble into vesicles in the presence of acrylate monomers. These vesicles encapsulate acrylate monomers and a photoinitiator within their hydrophobic region that can also accommodate a pore-forming template. (2) Vesicle extrusion through a track-etch membrane, followed by (3) UV polymerization of the monomer within the hydrophobic region. (4) To complete the synthesis process, the surfactant vesicle template and the pore-forming template are eliminated (Hambly et al., 2020). Copyright © 2020 American Chemical Society.

Furthermore, the exceptionally thin (∼1–5 nm) porous walls of hollow nanocapsules hold great promise for chemical and biosensor design. These thin, porous walls can be used as carriers for reagents in optical or electrochemical sensors and play a dual role by protecting the encapsulated contents and by facilitating the unimpeded bidirectional transport of analytes, substrates and reaction products. An innovative immobilization technique involves covalently bonding reagent-loaded nanocapsules to a conductive polymer poly(3,4-ethylenedioxythiophene) (PEDOT) film (Hambly et al., 2020). This approach, illustrated in Figure 6, overcomes the limitations associated with gel-like matrices, and its efficacy has been demonstrated by digital microscopy, scanning electron microscopy and depth-profiling X-ray photoelectron spectroscopy. This analysis confirmed the presence and distribution of nanocapsules within electrochemically deposited PEDOT films and also underscored the potential of this advanced immobilization strategy for revolutionizing sensor applications.

Overall, nanocapsule-based optodes are an innovative technology in ion detection, using nanocapsules as the sensing element. These optodes offer several advantages, including high sensitivity, selectivity, and stability (Kisiel et al., 2018). The encapsulation of ion-selective components within nanocapsules protects against environmental interference and enhances the optode’s performance in complex sample matrices. Additionally, nanocapsules can be engineered to release ionophores in response to specific stimuli, allowing for controlled and targeted ion detection. Moreover, the small size of nanocapsules enables their integration into various substrates and devices, expanding their applications in portable and miniaturized sensing platforms. However, challenges such as synthesis scalability, reproducibility, and biocompatibility need to be addressed for widespread adoption (Stelmach et al., 2020). To the best of knowledge, nanocapsules techniques was not used in the traditional way of PVC-membrane ion-selective optodes; instead of that, it was prepared using different polymers like Poly(maleic anhydride-alt-1-octadecene), poly(3,4-ethylenedioxythiophene), and others.

### 3.5 Nanoparticles that incorporate rare-earth-based optodes

Rare earth-based ISOs represent a new advance in ion-selective sensing, offering heightened sensitivity, selectivity and versatility for ion detection. The production process involves synthesizing luminescent materials from rare earth elements, integrating them into the optode matrix, and calibrating the system (Mahata et al., 2017). The optode was prepared in the traditional way using the chromoionophore and ionophore with rare-earth elements nanorods, all incorporated in the polymer thin film, which was used for the detection of pH and some metal cations (Xie et al., 2012). Nanorod-based optodes stand out for their innovative approach in which rare earth elements are used to enhance performance (Xie et al., 2012). Their fabrication includes synthesizing rare earth-doped nanorods with controlled size and shape using hydrothermal or solvothermal processes. Parameter tuning allows obtaining nanorods with specific dimensions and luminescent properties. The subsequent surface functionalization with ligands or ionophores enhances ion selectivity while preserving the luminescent properties. Functionalization involves chemically binding ligands or ionophores to the nanorod surface for stable attachment. Due to the unique optical characteristics of rare earth elements, these nanorods are promising materials for ion detection and quantification. Ongoing research focuses on refining the synthesis techniques, optimizing the calibration methods, and broadening the spectrum of detectable ions (Mahata et al., 2017).

A notable example of nanorod-based optodes is the work by Wu and Qin (2014) who used rare earth elements as precursors and oleic acid as a stabilizing agent. Nanorods (∼200 nm in diameter and 800 nm in length) were deposited on a quartz slide using a tetrahydrofuran solution that incorporated ionophores, chromoionophores and ion-exchangers. The authors tested this innovative approach for lead detection in industrial wastewater, demonstrating the practical applicability of rare-earth-based nanorods for environmental sensing. Rare earth-based optodes hold immense promise, and the current research focuses on improving the synthesis techniques to better control the nanorod properties and on functionalization strategies to enhance ion selectivity. However, challenges remain to be addressed, including optimizing the calibration methods and expanding the range of detectable ions (Mahata et al., 2017).

Overall, nanoparticles offer several advantages, including high stability, tunable optical properties, and resistance to photobleaching. The incorporation of rare-earth ions into the optode matrix enhances its sensitivity and selectivity, enabling precise detection of target ions in complex sample matrices. Additionally, the versatility of rare-earth elements allows for the engineering of nanoparticles with specific emission wavelengths, facilitating multiplexed detection of multiple ions simultaneously. Moreover, rare-earth-based optodes exhibit long luminescence lifetimes, enabling time-gated detection methods to minimize background noise and improve signal-to-noise ratios. However, challenges such as synthesis complexity, cost, and potential toxicity need to be addressed for practical applications.

## 4 Modern designs of ion-selective optodes

### 4.1 Disposable paper-based optodes

Disposable paper-based optodes, valued for their simplicity, cost-effectiveness and single-use convenience, are interesting for many applications, particularly in POC testing and environmental monitoring. These optodes can be used for pH measurement, where indicator dyes, such as bromothymol blue or phenolphthalein, impregnated onto paper strips, give a visual indication of the pH level, and are commonly employed in educational settings. Kenney et al. (2018) developed a pH-sensitive thin film optode to generate spatiotemporal pH gradient maps in tumour cell cultures using fluorescence microscopy. This device gives fast responses and is reversible and non-cytotoxic. Functionalization with specific ligands extends the utility of paper-based optodes to heavy metal ion detection. Phichi et al. (2020) fabricated a paper-based bulk optode for the concomitant optical determination of Hg^2+^ and Ag^+^, using benzothiazole calix (Hu et al., 2018) arene as highly selective ionophore. The sensor displays reproducible LODs for Hg^2+^ and Ag^+^, demonstrating the versatility of disposable paper-based optodes for targeted ion detection applications. Kassal et al. (2019b) proposed a method for potassium monitoring using PVC-membrane film that was deposited on paper substrate. The method was simple, of low cost, and was applied for potassium determination in the range of 10^−4 ^– 10^ −1^ M.

Paper-based optodes are extensively employed for environmental monitoring of ions (e.g., phosphate) in water samples. These optodes are based on ionophore-coated paper strips. Interaction with the target ions induces color or fluorescence changes that are correlated with the ion concentration. Bordbar et al. (2020) optimized a paper-based optode sensor that can detect and identify major organophosphate and carbamate pesticides with high selectivity and discrimination. In clinical diagnostics, Kitchawengkul et al. (2021) synthesized a microfluidic paper-based analytical device (µPAD) coupled with N-CQDs for the fast determination of total cholesterol in whole blood. Similarly, Gabriel et al. (2017) designed a paper-based glucose sensor for measuring glucose in human tears, providing results that are not significantly different from those of commercial glucose meters. Lookadoo et al. (2021)showed that a paper-based optode device with a smartphone optical reader could determine K^+^ in biological fluids with good dose-response linearity for POC monitoring. Moreover, Tan et al. (2020) developed a tape-paper-based 3D-microfluidic optode for the POC measurement of total bilirubin concentration in the blood of newborns.

Paper-based optodes find applications also in food safety testing. Hu et al. (2022) employed a paper-based optode for the visual detection of milk freshness based on the response of N-CQDs to milk acidity (described in Figure 4). Traditional paper-based analytical devices can be enhanced by incorporating microfluidic channels (µPADs) to control the liquid flow. After their first description by Martinez et al. (2007), in 2008 (REF), µPADs are now mainly based on colorimetry, offering simplicity and compatibility with smartphone-based systems. Shibata et al. (2019) developed a low-cost µPAD for the nano-optode-based Ca ion determination in water, demonstrating the potential of µPADs as cost-effective portable measuring devices. The authors have overcome the paper-based optodes requirement for specialized equipment or instruments for readout and analysis as they have relied on naked-eye detection of Ca ion concentrations.

Disposable paper-based optodes may have a limitation in terms of scalability as large-scale production can be challenging. However, the use of cellulosic filter papers as substrates for optodes combined with inkjet printing plays an important role in overcoming this problem. Briefly, polyvinyl chloride (PVC), ionophore, chromoionophore, ion-exchanger, and plasticizer are dissolved in an organic solvent to create particles suitable for deposition or inkjet printing on the filter paper (Soda et al., 2018). This preparation method allows the mass production of filter-based sensors with high reproducibility through inkjet deposition from aqueous dispersions, using conventional office inkjet printers. Wang et al. (2015) presented a plasticizer-free paper-based optode for sodium detection where the paper lipophilicity was exploited for optimal adsorption of the ionophore, chromoionophore, and ion-exchanger onto the cellulosic paper substrate. This plasticizer-free paper-based optode behaved like conventional optodes.

The choice of paper type influences the color intensity. For instance, Whatman^®^ filter paper grade 1 gives optimal results for glucose detection (Evans et al., 2014). Yang et al. (2013) developed a paper-based sensor using the Drabkin reagent for hemoglobin determination, achieving a LOD of 1 g/dL and a limit of quantification (LOQ) of 2.5 g/dL. Wirojsaengthong et al. (2021) used a paper-based optode to measure thiocyanate (a marker of active smoking) in urine samples, to distinguish between smokers and non-smokers, with LOD and LOQ values of 0.65 and 1.87 μmol L^−1^, respectively. Pena-Pereira et al. (2016) focused on instrumental-free determination and developed a paper-based optode for thiocyanate testing in saliva with LOD and LOQ values of 0.06 and 0.21 mmol L^−1^, respectively.

Overall, disposable paper-based optodes offer a unique and practical approach to ion detection, especially in resource-limited or field settings (Phichi et al., 2020). Unlike traditional optodes, these devices are typically fabricated on paper substrates, providing cost-effective and easily disposable platforms for ion sensing (Kassal et al., 2019a). One significant advantage is their simplicity and ease of use, making them accessible even to non-experts. Additionally, disposable paper-based optodes often exhibit rapid response times, enabling quick measurements in real-time (Kassal et al., 2019a). They are also lightweight and portable, making them ideal for on-site testing or remote monitoring applications. Furthermore, paper-based optodes can be easily integrated with other analytical techniques or devices, enhancing their versatility and functionality (Wang et al., 2015). However, one limitation is their relatively lower sensitivity and selectivity compared to some other optode types. Additionally, the fabrication process may require optimization to ensure uniformity and reproducibility across batches. Despite these challenges, disposable paper-based optodes offer a promising solution for point-of-care diagnostics, environmental monitoring, where simplicity, affordability, and portability are key considerations.

### 4.2 Smartphone-based optodes

Smartphone-based optodes epitomize the convergence of traditional optical sensing with contemporary smartphone technology to generate a portable and adaptable method for analytical measurements. These advanced tools exploit the smartphone inherent features (e.g., built-in cameras, computational capabilities and connectivity) to perform optical analyses. By incorporating specific recognition elements onto a sensor platform, smartphone-based optodes detect target analytes and generate optical signals captured by the smartphone camera. A key advantage lies in their accessibility and ease of use. As smartphones are ubiquitous worldwide, these optodes offer a democratized approach to analytical testing, eliminating the need of specialized equipment and technical proficiency. Users simply place their samples on the optode, capture images with the smartphone camera, and use dedicated mobile applications or software to analyze the optical signals and obtain quantitative results. This seamless integration of optical sensing and smartphone technology heralds a new era in analytical instrumentation, allowing users to perform sophisticated measurements with unprecedented convenience and simplicity.

**FIGURE 7:**
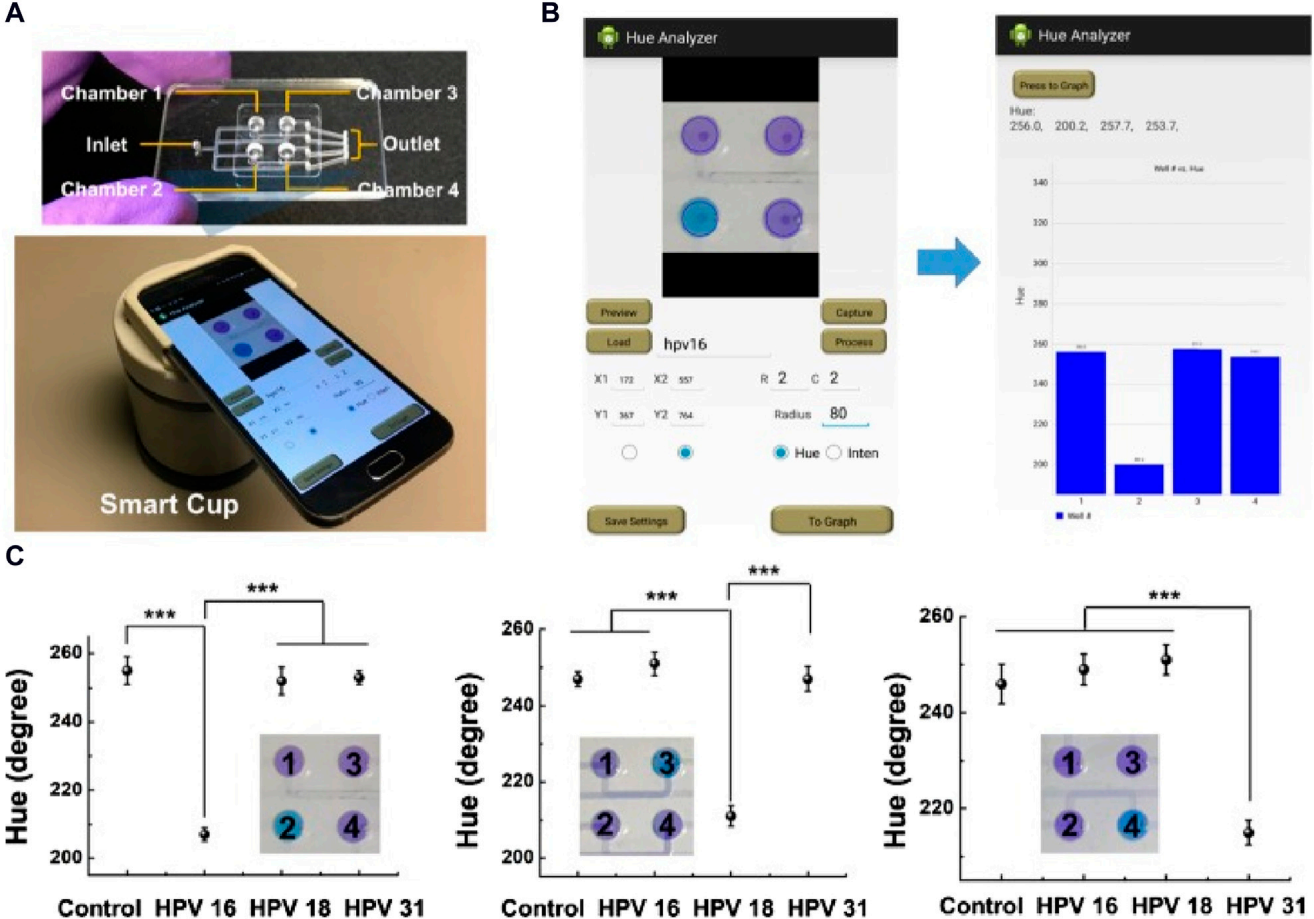
Smartphone-based POC optical device to detect HPV DNA in saliva samples. **(A)** The top panel shows the four-chamber microfluidic chip and the bottom panel the smartphone-equipped smart cup. **(B)** Photographs of the application user interfaces, including settings (left) and readout (right). **(C)** Detection of HPV DNA in spiked or not (control) saliva samples; ***p < 0.001 (*t*-test); error bars indicate the standard deviation (n = 3) (Yin et al., 2019). Figure reused from Yin et al., 2019 under the Creative Commons Attribution-NonCommercial (CC BY-NC) license.

Tian and collaborators (Tian et al., 2021) introduced an innovative smartphone-based device for visual and on-site detection of fluoride in groundwater. They designed a specific molecule, 2-(tert-butyl-diphenylsilanyloxy)-5-nitro-1H-benzoimidazole, that undergoes Si-O bond cleavage upon interaction with fluoride. This interaction induces a shift in the emission characteristics from an enol-like state at 437 nm to a keto-like tautomerism emission at 550 nm. This device displayed a low LOD (0.11 μM, 2.09 ppb) for fluoride with minimal interference. In another study, Yin et al. (2019) developed a disposable microfluidic chip tailored for colorimetric loop-mediated isothermal amplification (LAMP) to directly detect the DNA of different human papillomavirus (HPV) strains using a POC smart cup. The color signal from the LAMP assay is captured and analyzed using the “Hue Analyzer” Android application to increase the colorimetric readout precision, leading to a 10-fold increase in detection sensitivity. The effectiveness of this approach was demonstrated for HPV-related cancer screening using HPV-spiked saliva samples and clinical cervical swab specimens. Figure 7 shows the remarkable sensitivity of this groundbreaking smartphone-based method for HPV DNA detection (from 102 copies), a performance comparable to that of the currently used methodologies.

The global expansion of telecommunication infrastructure has significantly facilitated the field of analytical chemistry through the use of smartphones as detectors to quantify analytes for POC analyses and also for industrial, environmental and educational applications (Mudanyali et al., 2012; Shen et al., 2012; Zhu et al., 2012; Lopez-Ruiz et al., 2014; Xiao et al., 2020). This concept, initially emphasized by Mudanyali et al. for the rapid diagnosis (Mudanyali et al., 2012), extends to POC detection using the RGB system that allows obtaining reliable calibration curves and quantification of unknown samples. Due to its versatility, it is suitable for various optode systems, including paper-based setups (Mudanyali et al., 2012; Shen et al., 2012; Zhu et al., 2012). To the best of knowledge, smartphones detection tool was not applied for the PVC-membrane based-optodes, but it is mainly applied for paper-based optodes (Mudanyali et al., 2012; Shen et al., 2012; Zhu et al., 2012). For the colourimetric detection of Na, (Evans et al., 2014) employed smartphones and plasticizer-free paper, streamlining the preparation and measurement processes. Gavrilinko (Saranchina et al., 2021) demonstrated the smartphone-based quantification of fluoride in water with a detection limit below the spectrophotometer detection limit, for an analytical range from 0.1 to 30 mg L^−1^. For glucose concentration determination by a biosensing platform (Alizadeh et al., 2019), color changes were captured using a mobile camera and analyzed through a smartphone application. This setup allows the fast and sensitive quantification of glucose with a LOD of 25 μM. Guzman et al. (2018) integrated μPADs in a portable detection system for formaldehyde (CH_2_O) detection. This device surpassed the LOD of the standard spectrophotometric method. As researchers explore advancements in smartphone technology, including improved camera quality, augmented reality and artificial intelligence, smartphone-based optodes hold the potential to revolutionize analytical measurements, by offering accurate and rapid testing in a portable format.

Overall, smartphone-based optodes offer an innovative and accessible approach to ion detection, capitalizing on the widespread availability and advanced capabilities of smartphones (Cui et al., 2022). Their primary advantage lies in their convenience and portability, facilitating on-the-go testing across diverse environments. Moreover, these optodes often harness the computational power of smartphones for real-time data analysis, enabling instant results interpretation and sharing. This capability renders them particularly valuable for point-of-care diagnostics, and environmental monitoring. Additionally, smartphone-based optodes seamlessly integrate with other smartphone functionalities, such as GPS and internet connectivity, facilitating remote data collection and sharing. However, a key limitation is the necessity for calibration and validation to ensure measurement accuracy and reliability. Furthermore, the performance of smartphone-based optodes may fluctuate based on factors like the quality of the smartphone camera or sensor and ambient lighting conditions. Despite these challenges, the accessibility, affordability, and versatility of smartphone-based optodes make them a promising tool for democratizing ion detection. Their potential to expand ion detection applications to a broader audience holds significant promise, empowering individuals across various fields to engage in ion detection tasks efficiently and effectively.

### 4.3 Wearable ion-selective optodes

Wearable ISOs are innovative devices that merge ISOs with wearable technology to enable real-time, non-invasive monitoring of specific ions directly on the human body. This innovation marries the principles of ion-selective sensing with the portability and convenience of wearables, introducing a new era in personal health monitoring and environmental assessment. Their design incorporates crucial components, such as ionophores and chromoionophores, into wearable platforms (e.g., wristbands, patches or textiles) (Koh et al., 2016). These components interact with target ions in the environment or body fluids, inducing observable changes in optical properties that are quantified and transmitted to a device (e.g., smartphone). Wearable ISOs have many applications, from fitness tracking to healthcare and environmental monitoring. In fitness tracking, they can continuously monitor the electrolyte levels in sweat during physical activities, offering information on the hydration status (Kassal et al., 2019a). In healthcare, wearable ISOs can track ions that are important for health, such as glucose and Ca (You et al., 2023). Due to their unobtrusive design and wireless connectivity, they are easily integrated into the daily routines, facilitating proactive health management and informed decision-making based on real-time data (Yang and Gao, 2019). As wearable technology advances, wearable ISOs hold the potential to revolutionize personalized health and environmental monitoring, advancing our understanding of ion dynamics in real-world contexts.

**FIGURE 8:**
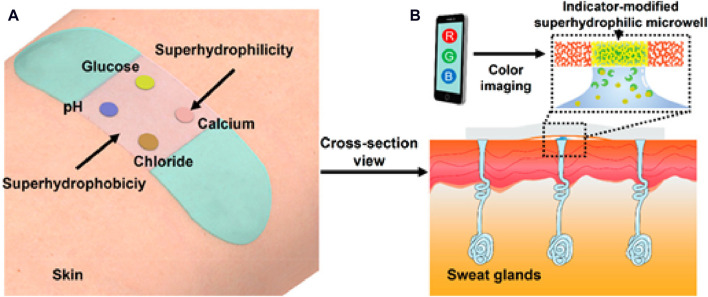
Paradigmatic example of a wearable optical sensor designed for real-time monitoring of sweat. **(A)** Wearable band seamlessly integrated onto the skin for continuous sweat monitoring. **(B)** Cross-sectional view showing the efficient pumping of sweat from sweat glands into indicator-modified super-hydrophilic microwells. Sweat samples are analyzed using a cellphone-assisted RGB screening system (He et al., 2019). Copyright © 2019 American Chemical Society.

Kassal et al. (2019b) developed a traditional type membrane optode using PVC polymer, and demonstrated the continuous sweat monitoring by transferring this membrane components on a paper; this group reported the potential of application of this system in wearable sweat analysis. He and colleagues (He et al., 2019) developed a flexible and skin-mounted band designed to seamlessly incorporate superhydrophobic-superhydrophilic microarrays with nanodendritic colorimetric biosensors for real-time on-site sweat sampling and analysis (Figure 8). This pioneering development offers an independent platform dedicated to sweat bio-detection that relies on a smartphone-based analysis to assess various parameters, such as pH, chloride, glucose, and Ca levels. The integration of these wearable biosensors, embedded in super-wettable bands with enhanced interface control, holds immense promise for transforming sweat sampling at specific anatomical sites. This advancement significantly contributes to the feasibility of straightforward and non-invasive bio-fluid analyses, a crucial step towards personalized health monitoring and POC testing. The incorporation of different sensing modalities within a wearable, skin-mounted band enhances the versatility of real-time analysis, opening avenues for continuous health monitoring and providing valuable insights into an individual’s health status. The potential applications of this technology are extensive, from athletic performance optimization to disease management through personalized data-driven interventions.

Overall, wearable ion-selective optodes are a groundbreaking innovation in ion detection technology, designed to be worn directly on the body for continuous monitoring of ions in real time (Li et al., 2021). One significant advantage is their non-invasive nature, allowing for comfortable and convenient monitoring without the need for invasive procedures. These optodes offer the potential for continuous, unobtrusive monitoring of ions such as sodium, potassium, or glucose, making them invaluable for healthcare applications like monitoring electrolyte levels or managing chronic conditions. Additionally, wearable ion-selective optodes can be integrated into wearable devices such as smartwatches or fitness trackers, leveraging existing technology for seamless data collection and analysis. This integration enhances their accessibility and usability, enabling individuals to monitor their ion levels conveniently throughout the day. However, challenges include ensuring accuracy and reliability in dynamic environments and optimizing power consumption to prolong battery life (Min et al., 2021).

Table 1 provides a comprehensive summary of novel and modern ISO designs, listing the various substrates, target analytes, coloring agents, concentration ranges, and detection limits. For instance, using polystyrene microspheres, ISOs were developed for K^+^ and salicylate detection at concentration ranges of 10^–6^–10^–1^ M and 3–70 μM, respectively (Xie et al., 2014a; Abdel-Haleem and Zahran, 2019). Polymer dots were employed for the detection of glucose (from 5 to 18 mM) with a LOD of 5 mM (Sun et al., 2018). ISOs are very versatile and can be used to monitor many different analytes, including metal ions (Cu^2+^, Hg^2+^), pH and biomolecules (glucose). Additionally, advanced designs involve wearable technology, such as a non-invasive biosensor incorporated into a Samsung Gear 2STM smartwatch for glucose detection in sweat (Rodin et al., 2019) and an ion-selective wearable skin-mounted band that can monitor pH, Cl^−^, glucose, and Ca^2+^ in sweat (He et al., 2019). These innovations underscore ISO evolving landscape for a wide range of applications, from environmental monitoring to healthcare and wearable technology.

**TABLE 1:** Summary of some novel and modern designs of ion-selective optodes.

Optode substrate	Analyte	Coloring agent	Concentration range	Detection limit	Ref.
Polystyrene microspheres	K^+^	Chromoionophore I	10^–6^–10^–1^ M	10^–6^ M	Xie et al. (2014a)
Polystyrene microspheres	Salicylate	ETH 7075	3–70 µM	2.1 µM	Abdel-Haleem and Zahran (2019)
Pluronic F-127 nanospheres	Ca^2+^	Chromoionophore I	10^–7^–10^–5^ M	10^–7^ M	Xie et al. (2014b)
B,N-GQDs dispersed in an aqueous solution	Hg^2+^ in real water samples	Fluorescence quenching	0.2–1 µM	6.5 nM	Liu et al. (2020)
N,S-GQDs dissolved in water	Hg^2+^ in living cells	Fluorescence quenching	0.9–30 nM	0.69 nM	Qu et al. (2019)
Polymer dots	Glucose	PD4Gx (Pd-porphyrin-based transducer)	5–18 mM	5 mM	Sun et al. (2018)
Semiconducting polymer dot (PFO–PFPV Pdot)-based fluorescent probe	Hypochlorous acid in living cells	Oxidative quenching	0.05–3.5 µM	56.8 nM	Zhu et al. (2021)
Dye-loaded porous nanocapsules in a hydrogel matrix	pH	Nile blue A	7.5–9.3 pH unit	±0.03 pH unit (resolution)	Maclin et al. (2015)
Colloidal solution of silver nanocapsules (AgNCs)	Cu^2+^	Redox reaction	0–400 nM	2.6 nM	Vellaichamy and Periakaruppan (2017)
Rare-earth metal (Er, Yb) upconverting nanorods	pH Na^+^ Ca^2+^ K^+^ Cu^2+^	ETH 5418	6–9 pH units 10^–5^–10^–2^ M (Na^+^) 10^–7^–10^–4^ M (K^+^) 10^–6^–10^–3^ M (Cu^2+^)	Not stated	Xie et al. (2012)
Lanthanide functionalized metal-organic frameworks by encapsulating Eu^3+^ cations in the pores of MIL-53–COOH (Al) nanocrystals	Intracellular Fe^3+^	Luminescence quenching	0.5–500 µM	0.5 µM	Zhou et al. (2014)
Disposable cellulose paper	Ag^+^ Hg^2+^ in water	Chromoionophore XIV	1.92 × 10^−6^–5 × 10^−3^ M (Ag^+^) 5.74 × 10^−7^–5 × 10^−5^ M (Hg^2+^)	1.92 × 10^−6^ M (Ag^+^) 5.74 × 10^−7^M (Hg^2+^)	Phichi et al. (2020)
Disposable chromatography paper	Glucose in tears	3,3′,5,5′-tetramethylbenzydine	0.1–1 mM	50 µM	Gabriel et al. (2017)
Spot plate integrated into smartphone-based optodes	F^−^ in groundwater	2-(tert-butyl-diphenylsilanyloxy)-5-nitro-1H-benzimidazole	1–14 µM	0.11 µM	Tian et al. (2021)
Non-invasive biosensor incorporated into a Samsung Gear 2S™ smartwatch	Glucose in sweat	Optical properties of the smartwatch	60–80 mg/dL, and ˃200 mg/dL (comparable to that of a commercial glucometer)	Not stated	Rodin et al. (2019)
Ion-selective wearable skin-mounted band	pH Cl^−^ glucose Ca^+^ in sweat	Litmus solution (pH) Mercuric thiocyanate (Cl^−^) KI (glucose) o-cresolphthalein complexone method (Ca^2+^)	4.5–7 (pH) 0–100 mM (Cl^−^) 0–15 mM (glucose) 0–15 mM (Ca^+^)	-	He et al. (2019)

## 5 Conclusion and remarks

In the rapidly evolving landscape of ISO, remarkable advances have been described in recent years, driven by inventive designs and the integration of nanomaterials. ISOs exhibit remarkable features for various applications. Moreover, addressing their traditional type and its mechanism, inherent challenges and constraints will inspire future advances. Achieving high selectivity in complex sample matrices is one of these challenges because interference from other ions can compromise accuracy. For instance, in environmental monitoring, ISOs may encounter ions with similar properties. Therefore, innovative strategies are required to enhance their specificity. Another limitation is their sensitivity range. Detecting trace ion concentrations can be challenging due to the limitations of signal transduction mechanisms. Color-based changes may not offer the required sensitivity in the presence of ultra-low ion concentrations. Striving for increasing sensitivity without sacrificing selectivity remains an ongoing endeavor. Stability and durability are another issue, especially when such devices are used repeatedly or in harsh conditions. Moreover, optode components can undergo degradation and this will affect their accuracy and longevity. For instance, in medical diagnostics, optodes used internally must endure harsh conditions. Developing more resilient materials and protective coatings could extend their lifespans. Future opportunities lie in nanotechnology and materials science. Nanomaterials, such as QDs, nanoparticles and nanocomposites, offer increased surface area and transduction capabilities, giving the possibility to fabricate ultra-sensitive and multiplexed sensors. Additionally, artificial intelligence algorithms can enhance data analysis, enabling real-time monitoring and pattern recognition for swift and accurate ion detection. Miniaturization and integration in wearable devices or microfluidic systems are promising approaches. They could lead to the development of portable sensors for continuous monitoring in various fields, including healthcare, agriculture, and industry. In conclusion, ISOs have achieved commendable progress, but challenges persist in selectivity, sensitivity, stability, and miniaturization. Addressing these issues and exploiting new avenues (nanotechnology and miniaturization) will pave the way to more sophisticated and adaptable ion detection systems for many different applications.
